# Editorial: Human Exposure to New-Emerging Electric, Magnetic and Electromagnetic Fields

**DOI:** 10.3389/fpubh.2022.894624

**Published:** 2022-04-28

**Authors:** Tongning Wu, Ruiyun Peng, Lei Zhang, Kun Li

**Affiliations:** ^1^China Academy of Information and Communications Technology, Beijing, China; ^2^Beijing Institute of Radiation Medicine, Academy of Military Medical Sciences, Beijing, China; ^3^Department of Occupational Health, Third Military Medical University, Chongqing, China; ^4^Faculty of Engineering and Design, Kagawa University, Takamatsu, Japan

**Keywords:** terahertz, millimeter wave, microwave, radiofrequency, low-frequency

With the advancement of wireless technologies and electronic/electrical devices, humans are exposed to more complicated electric, magnetic, and electromagnetic fields (EMF), which has raised public concerns on potential health effects. Researchers have recently conducted a series of studies on diverse exposure scenarios. In addition, international standard organizations have revised safety guidelines and standards (1). These recent results and practices can enhance our knowledge in assessing health risks from the exposure to EMF (2). This Research Topic consists of 14 articles (one review article, two brief research report articles, and 11 original research articles) published in the Radiation and Health section of Frontiers in Public Health.

There have been immense concerns over the neurological effect of EMF exposure. Hu et al. reviewed the effects on metabolism and receptors of neurotransmitters in the brain and found them to be important, while the mechanisms have not yet been clarified. The authors advocated the application of novel techniques, such as neuroviral tracers, neuroimaging, and neuroelectrophysiology. Li et al. investigated the effects of 1,800 MHz EMF exposure for 48 h on neurite outgrowth in neuronal cells and explored the associated role of the Rap1 signaling pathway, using primary hippocampal neurons from C57BL/6 mice and Neuro2a cells. They found that the neurite length, primary and secondary neurite numbers, and branch points of primary mouse hippocampal neurons were significantly impaired by 48-h RF-EMF exposure. The neurite-bearing cell percentage and neurite length of Neuro2a cells were inhibited by 48-h RF-EMF exposure. Rap1 activity was inhibited by 48-h RF-EMF with no detectable alteration in either gene or protein expression of Rap1. The protein expression of Rap1GAP increased after 48-h exposure, while the expression of p-MEK1/2 protein decreased. Concerning human studies, Yang et al. conducted functional magnetic resonance imaging for 17 adults. No alteration was found before and after exposure to a 30-min time-division long-term evolution (LTE) signal (2.573 GHz) with an intensity similar to the maximum emission of a mobile phone. The authors generated intrinsic connectivity networks and conducted static and dynamic functional network connectivity analysis. There exists very few human studies using functional magnetic resonance imaging. The results were generally consistent (3) but the scarce number of the subjects may be a limitation of these articles. These pilot publications shed light on defining the exposure protocol and selecting the appropriate neurophysiological/dosimetric metrics for future human studies.

There is a revival in the interest of revisiting the exposure that people experience every day. A pulsed low-frequency magnetic field is widely generated by household appliances and the potential biological effect on fetuses has attracted public concerns. Sun et al. investigated the effect of 10 Hz pulsed magnetic field exposure on cellular senescence in a 2BS cell line, isolated from human fetal lung fibroblasts, and found that 1 day on/1 day off exposure to the magnetic field of 1.0 mT for 2 weeks induced DNA damage, but no other significant phenotype of cellular senescence in the cells. Xie et al. found the exposure to low-dose 900 MHz signals increased the level of reactive oxygen species and activated a mitochondrial unfolded protein response in mouse bone marrow stromal cells. The increased HSP10/HSP60/ClpP protein level lasted up to 4 h, while mitochondrial homeostasis was restored 24 h after exposure. However, the molecular mechanism was unclear.

New exposure scenarios posed challenges to quantify the EMF distribution which would make the result reproducible. Wang et al. assessed the dosimetric variability of the free rats in exposure experiments of 1.8 GHz. Chakarothai et al. proposed a frequency-dependent finite-difference time-domain method for human exposure to pulsed EMF at microwave bands. The use of a four-term Cole-Cole model for complex permittivity functions, applying the fast inverse Laplace transform and Prony method, improved the accuracy when calculating the specific energy absorption for biological tissues. To characterize exposure by 4G signals, Mazloum et al. conducted measurement campaigns in a multi-floor indoor environment using a drive test solution to record both downlink (DL) and uplink (UL) connection parameters for LTE networks. Several typical usage services were involved and investigated. They proposed an artificial neural network model to accurately estimate UL TX power with a mean absolute error of 1.487 dB. Concerning 5G, Xu et al. reported the computational results of actual maximum EMF exposure and the corresponding power reduction factors (PRFs) for millimeter-wave (28 GHz) base station antennas. They clarified the effectiveness of using this far-field approach, which can also guarantee the conservativeness of the PRFs for the assessment of the actual maximum exposure close to the antenna. A 60 GHz frequency point could also be deployed by 5G. Hikage et al. developed an exposure setup for the experiment of biological effects of local exposure to millimeter waves on the human body. The designed system enabled the researchers to achieve high-intensity 60 GHz irradiation to the target area of the human body by a spatial synthetic beam-type exposure setup with two dielectric lens antennas. Interference fringes can be generated in the exposed area by applying an orthogonalizing polarized feeding structure, where the expected local temperature changes at the target area of the human forearm skin were confirmed. Li et al. reported a formula-based analysis of cloth effects on human skin exposure to obliquely incident electromagnetic waves at 60 GHz by the Monte Carlo method. The analyzed absorbed power density (APD) within the skin surface covered by different cloth materials increased up to 40% compared with that of bare skin, but all the results of the APD did not exceed the basic restriction for local exposure specified in the exposure guidelines and standards. In particular, occupational exposure in a military scenario was accessed by Colella et al. The obtained E-field values radiated in the free space by a HF vehicular antenna may exceed the safety guideline reference levels. However, SAR and E-field values induced inside the body remained well below safety limits.

There were also two studies dedicated to exposure of medical devices. RF-induced heating in implantable devices during magnetic resonance imaging is a complex function of many different clinical factors. To reduce implant heating and maintain good image quality at the same time, Yao et al. developed an exposure optimization trail that allowed for comprehensive optimization in an efficient and traceable manner. Major clinical factors were decoupled from the redundant dataset using principal component analysis. Exposure optimization for a 40-cm cardio implant was demonstrated with the proposed workflow. Lu et al. optimized the position of a transcranial magnetic stimulation coil to maximize the E-field distribution in the target brain region and also reduced unnecessary exposure to the other regions. A genetic algorithm was applied in this practice relating to stroke.

The editors of this Research Topic are grateful to all distinguished authors for their invaluable contributions and are indebted to the expert reviewers for their time, dedication, and constructive comments. We wish to acknowledge the Radiation and Health section of Frontiers in Public Health for the opportunity to guest edit this Research Topic.

## Author Contributions

TW and KL drafted the editorial and RP and LZ commented on it. All authors contributed to the guest editing of the Research Topic.

## Conflict of Interest

The authors declare that the research was conducted in the absence of any commercial or financial relationships that could be construed as a potential conflict of interest.

## Publisher's Note

All claims expressed in this article are solely those of the authors and do not necessarily represent those of their affiliated organizations, or those of the publisher, the editors and the reviewers. Any product that may be evaluated in this article, or claim that may be made by its manufacturer, is not guaranteed or endorsed by the publisher.
